# Noncoding RNAs Involved in the Pathogenesis of Ankylosing Spondylitis

**DOI:** 10.1155/2019/6920281

**Published:** 2019-07-07

**Authors:** Chong Chen, Tianhua Rong, Zheng Li, Jianxiong Shen

**Affiliations:** Department of Orthopaedic Surgery, Peking Union Medical College Hospital, Chinese Academy of Medical Sciences, Peking Union Medical College, Beijing, China

## Abstract

Ankylosing spondylitis (AS) is a form of arthritis that can lead to fusion of vertebrae and sacroiliac joints following syndesmophyte formation. The etiology of this painful disease remains poorly defined due to its complex genetic background. There are no commonly accepted methods for early diagnosis of AS, nor are there any effective or efficient clinical treatments. Several noncoding RNAs (ncRNAs) have been linked to AS pathogenesis and inflammation via selective binding of their downstream targets. However, major gaps in knowledge remain to be filled before such findings can be translated into clinical treatments for AS. In this review, we outline recent findings that demonstrate essential roles of ncRNAs in AS mediated via multiple signaling pathways such as the Wnt, transforming growth factor-*β*/bone morphogenetic protein, inflammatory, T-cell prosurvival, and nuclear factor-*κ*B pathways. The summary of these findings provides insight into the molecular mechanisms by which ncRNAs can be targeted for AS diagnosis and the development of therapeutic drugs against a variety of autoimmune diseases.

## 1. Introduction

Ankylosing spondylitis (AS) is a type of arthritis most commonly affecting the spine that is characterized by back pain, syndesmophyte formation, fusion of the spine and sacroiliac joints, and disability. In AS, inflammation in various regions of the skeletal system induces new bone formation [1].

Through transcriptional analysis and RNA-labeled sequencing, the Australo-Anglo-American Spondyloarthritis Consortium has identified all of the RNA labels from each long noncoding RNA (lncRNA; >200 nucleotides) transcript isolated from AS-associated gene deserts in peripheral blood monocytes [2]. Now further research is needed to explore the roles of the identified ncRNAs in the pathogenesis of AS. Moreover, analysis of sequence variation in ncRNAs has never been properly conducted to determine independent risk factors for AS among those common for human diseases. Recently, the contributions of microRNAs (miRNAs), a subfamily of small ncRNAs that regulate target gene transcripts, to autoimmune diseases have been recognized [3]. These miRNAs target several signaling pathways that are potentially involved in the specific mechanisms underlying AS pathogenesis, whereas lncRNAs have been reported to have important roles in major cellular processes such as chromatin remodeling and subsequent transcription, posttranscriptional processing, and integrity of the nucleus [4–7]. Ultimately, lncRNAs, through various functions, regulate cellular proliferation, migration, and differentiation as well as organogenesis [8].

Furthermore, previous research suggests that lncRNAs maintain body homeostasis and contribute to rheumatic and autoimmune diseases.

**Figure 1:**
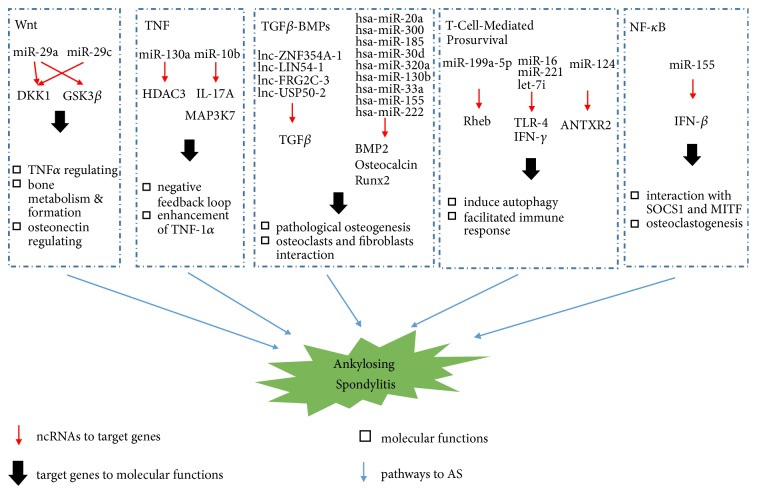
Functional roles of specific ncRNAs in the pathogenesis of AS. Red arrow: ncRNAs point to target genes; black arrow: target genes point to molecular functions; black square: molecular functions; blue arrow: signaling pathways point to AS.

In the present review, we summarize the literature describing the emerging roles of ncRNAs in multiple signaling pathways that contribute to several autoimmune diseases, with an emphasis on AS (Tables 1 and 2). We also illustrate the role of ncRNAs in the pathogenesis of AS (Figure 1). Specifically, we discuss the function of ncRNAs in the Wnt, transforming growth factor-*β* (TGF*β*)/bone morphogenetic protein (BMP), inflammatory, T-cell prosurvival, and nuclear factor-*κ*B (NF-*κ*B) signaling pathways. ncRNAs are capable of targeting members of these signaling pathways, and we suggest that ncRNAs represent molecular targets for AS diagnosis and the development of therapeutic drugs against autoimmune diseases.

## 2. Wnt Signaling Pathway

Wnt signaling is evolutionarily conserved in metazoans and essential for cellular processes including cell fate determination, migration, and polarization as well as neural patterning, organogenesis, tissue homeostasis, and tissue repair after stroke or traumatic injury [28, 29]. Accumulating evidence indicates that Wnt signaling has clinical implications in the pathogenesis of autoimmune diseases, including AS,* via *its role in bone morphogenesis and homeostasis through the induction of mesenchymal cell osteoblastogenesis. This process may be an important contributor to the anabolic metabolism involved in joint remodeling in patients with AS and/or osteoarthritis [30].

Dickkopf-1 (DKK1), a potent inhibitor of the canonical Wnt signaling pathway, is required during Drosophila embryonic head development and interacts with its coreceptor LRP5. LRP5 dysfunction and/or deficiency can result in erosive arthritis [9]. Additionally, some studies have reported that promotion of Wnt/*β*-catenin signaling, e.g., by antagonism of DKK1, is a promising therapeutic option for patients with bone pathologies [31].

DKK1 is considered a biomarker for the early detection of new bone formation in AS patients [32, 33]. However, another study reported a compensatory increase in circulating endogenous factors that promote new bone formation, which they attribute to a decrease in DKK-1-mediated inhibition in AS patients [34]. Huang* et al. *[10] reported higher expression of miR-29a in AS patients than in patients with rheumatoid arthritis (RA) or healthy individuals, which suggests the potential of miR-29a as a novel biomarker for AS. Functionally, one study showed that miR-29a mediates tumor necrosis factor-*α* (TNF*α*)-induced bone loss via selective suppression of DKK1 and glycogen synthase kinase 3*β* (GSK3*β*), which activates the canonical Wnt/*β*-catenin signaling pathway [9]. Nevertheless, increased miR-29a expression was not found to correlate with the Bath Ankylosing Spondylitis Functional Index (BASFI), an index that reflects the degree of functional limitations in AS patients. Moreover, the expression levels of both miR-29a and miR-29c were decreased in osteoblasts following treatment with DKK-1, revealing that miR-29 expression is induced by an increase in canonical Wnt signaling during osteoblastic differentiation [11]. In brief, these results indicate that miR-29a is essential for the regulation of TNF*α*-mediated osteogenic differentiation, at least in part through the regulation of DKK1 and GSK3*β*, and thereby enhances canonical Wnt signaling [9].

## 3. TNF Signaling Pathway

Over the last two decades, the use of TNF inhibitors has improved AS treatment by reducing inflammation, leading to significant reductions in clinical symptoms in AS patients that were not achieved with conventional treatments.

TNF inhibitors have a recognized ability to inhibit spinal radiographic progression in AS patients [35, 36]. We hypothesize that this process may occur through the interaction of these inhibitors with DKK-1, given that neutralization of DKK-1 with antibodies has been widely used to reverse bone-destructive patterns in mouse models of RA, inevitably resulting in hyperosteogeny in patients with osteoarthritis [37]. In addition, Adalimumab, a monoclonal antibody to human TNF*α*, is an effective anti-inflammatory agent in AS patients, but its effect is also accompanied by reduced expression of DKK-1 in serum and increased focal fat deposition in the lumbar spine [38]. We hypothesize that the TNF-induced release of DKK1 inhibits Wnt signaling, which in turn reduces osteoblastogenesis and osteoprotegerin (OPG) expression and instead increases osteoclast activity and erosion. This is consistent with the observed reductions in serum DKK1 levels in RA patients treated with TNF inhibitors [37]. Furthermore, a study in a genetically engineered AS mouse model showed that treatment with DKK1-blocking antibodies promoted fusion of the sacroiliac joints [39].

Histone acetyltransferases (HATs) and histone deacetylases (HDACs), the master regulators of acetylation and deacetylation, respectively, modulate the expression levels of various genes, including those that encode inflammatory cytokines [40]. HDAC inhibitors have been administered to reduce the release of proinflammatory cytokines such as TNF*α* [41], which in turn reduces the expression of SIRT1 that is activated by the TNF*α*/NF-*κ*B signaling pathway [42]. Recently, HDAC3 was reported to modulate a negative feedback loop involving miR-130a expression through the upregulation of TNF*α* in peripheral blood monocytes, suggesting a potential molecular mechanism underlying the pathology of AS [12]. Further, TNF*α* upregulates miR-10b, which in turn suppresses IL-17A production, revealing an essential role for miR-10b in a negative feedback loop that inhibits the Th17-mediated inflammatory response in AS [43].

IL-1R-associated kinase (IRAK1) is a member of the signaling cascade that induces TNF expression and plays a critical role in providing negative feedback for TNF*α*-induced inflammation. IRAK1 is regulated by miR146a, a miRNA that inhibits osteoclastogenesis and has received attention for its potential use in the diagnosis of juvenile idiopathic arthritis [23, 44, 45].

## 4. TGF***β***-BMP Signaling Pathway

The BMPs, members of the TGF*β* superfamily, are released by various cell types including osteoblasts, chondrocytes, and endothelial cells [46]. Proosteogenic BMPs such as BMP2/4/7 bind cognate membrane-bound receptors, and these binding events induce phosphorylation of SMAD1/5/8, respectively. Phosphorylated SMAD1/5/8 then associate with SMAD4 and translocate to the nucleus to initiate transcription of BMP-responsive genes. Several secreted oligopeptides, such as Noggin (NOG) and Sclerostin, sequester BMPs via a competitive inhibition approach and prevent binding with TGF*β* receptors [13]. In the clinic, dysregulation of BMP signaling pathway is correlated with several skeletal disorders, including low- or high-bone mass diseases, heterotopic ossification, and osteoporosis [46].

BMP signaling is involved in the pathological osteogenesis that occurs in AS [47]. An imbalance between BMP2 and NOG contributes to BMP2-mediated increases in osteogenic differentiation of mesenchymal stem cells in AS patients. Xie* et al*. [14] performed microarray analyses with mesenchymal stem cells from both AS patients and healthy control individuals after 10 days in conditions promoting osteogenic differentiation. Their findings demonstrated differences in lncRNA and mRNA expression between these two groups. For example, analysis of coexpression networks, also known as protein-coding and noncoding gene expression analysis, revealed that several lncRNAs, including lnc-ZNF354A-1, lnc-LIN54-1, lnc-FRG2C-3, and lnc-USP50-2, contribute to osteogenesis in AS patients. Furthermore, bioinformatic predictions of a number of miRNAs from* Homo sapiens *suggest a suppressive influence on several genes such as osteocalcin, BMP2, and Runx2, which are generally related to osteogenic differentiation. Those* Homo sapiens *microRNAs (hsa-miRs) include hsa-miR-20a, hsa-miR-30d, hsa-miR-33a, has-miR-130b, hsa-miR-155, hsa-miR-185, hsa-miR-222, hsa-miR-300, and hsa-miR-320a. Additionally, another study demonstrated that osteoclasts could induce the osteogenic differentiation of fibroblasts* in vitro*, and therefore, miRNAs may play key roles via their modulation of cellular interactions between osteoclasts and fibroblasts [15].

## 5. T-Cell-Mediated Prosurvival Signaling Pathway

Wang* et al. *[16] observed significant downregulation of autophagy-related genes such as LC3, Beclin1, ATG5, and miRNA-199a-5p in T cells of AS patients. They also observed higher concentrations of TNF*α*, interleukin (IL)-17, and IL-23 in the serum of AS patients relative to healthy persons. When Rheb, a known target of miRNA-199a-5p, was inhibited, strikingly different outcomes were observed due to the loss of Rheb-induced inactivation of the phosphorylating mechanistic target of rapamycin (mTOR), and the result was enhanced T-cell autophagy. Thus, miRNA-199a-5p overexpression represents a potentially useful therapeutic strategy for enhancing autophagy and inhibiting the pathogenesis of AS based on the selective modulation of Rheb expression and mTOR signaling.

Additionally, proinflammatory cytokines upregulate miR-10b expression, which functions as a negative autocrine/paracrine feedback inhibitor of IL-17A expression via interaction with MAP3K7. We posit that miR-10b is a potential therapeutic agent for AS treatment according to its ability to suppress pathogenic Th17 cell function, but further research focused on miR-10b is needed [43].

Among several ncRNAs, such as miR-16, miR-221, and let-7i, that are upregulated in T cells of AS patients, a recent study using the Bath Ankylosing Spondylitis Radiology Index (BASRI) test in the lumbar spine of AS patients revealed that miR-221 and let-7i are correlated with alterations in the BASRI [17]. Moreover, increased let-7i expression may facilitate the induction of interferon (IFN)-*γ* release by T helper type 1 cells, which facilitates immune responses [17]. Furthermore, following the overexpression of let-7i* in vitro*, insulin-like growth factor 1 receptor (IGF1R) expression is significantly decreased to levels similar to those observed with IGF1R siRNA treatment in Jurkat cells. Inhibition of IGF1R-mediated signaling reduces phosphorylation of mTOR/Akt and Bcl-2 expression levels and reduces negative modulation of Bax/caspase-3/PARP, thereby subsequently inducing autophagy. Similarly, let-7i overexpression induces autophagy, thereby protecting against T-cell apoptosis. This study revealed that let-7i is involved in the cellular decision-making process during the apoptosis/autophagy paradox via selective modulation of IGF1R in T cells from AS patients [48]. Additionally, genetic polymorphisms of IL-1R-associated kinase (IRAK1) have been correlated with AS susceptibility and predicted to impact miR-146's actions in the 3' untranslated region; however, no alteration of the miR-146a rs2910164 distribution was observed in AS patients [23]. Th1 cell differentiation and activation are considered to be, at least partially, mediated by the anthrax toxin receptor 2, ANTXR2 [49], which is selectively targeted by miR-124, and overexpression of ANTXR2 induces autophagy during bacterial infection in patients with AS [18].

## 6. NF-***κ***B Signaling Pathway

Receptor activator of NF-*κ*B ligand (RANKL) functions synergistically with proinflammatory cytokines to promote monocyte differentiation into osteoclasts in synovial tissues [50]. Inflamed synovial tissues contain lymphocytes and fibroblast-like synoviocytes that produce RANKL under various pathological conditions. miR-155, a member of the IFN-*β*-induced miRNAs, mediates the suppression of IFN-*β* in osteoclast differentiation through selective interaction with two proteins, suppressor of cytokine signaling 1 (SOCS1) and microphthalmia-associated transcription factor (MITF), that are critical regulators of osteoclastogenesis [19]. Upregulation of miR-155 leads to increases in the release of proinflammatory cytokines IL-6 and IL-23, which promote the maturation of autoreactive Th17 cells and release of TNF*α*, a well-established mediator of chronic inflammation [51]. Furthermore, miR-155 expression is closely correlated with the BASFI as well as severity indices of thoracolumbar kyphosis secondary to AS [20].

Upregulation of miR-146a has been reported in various autoimmune diseases such as psoriasis, RA, lupus nephritis, and Sjögren's syndrome [52–55]. Xu* et al*. [24] discovered a correlation between the miR-146a SNP rs2910164 and AS in Chinese patients, and Qian* et al*. [20] also reported that miR-146a is a novel biomarker for AS. However, when Niu* et al*. [25] conducted a frequency analysis of three common SNPs in miR-146a, i.e., rs2431697, rs2910164, and rs57095329, in Chinese AS patients, no significant correlations were observed between these three SNPs and AS. However, their findings still indicated the potential involvement of these SNPs in the progression of certain autoimmune diseases and even their potential value in corresponding treatment strategies. Moreover, miR-146a acts as an effective and efficient modulator to prevent overwhelming inflammatory reactions. Reductions in miR-146 expression contribute to the prolonged release of inflammatory cytokines that occurs after the NF-*κ*B signaling pathway is activated by lipopolysaccharide and other proinflammatory mediators [24, 56]. Two downstream targets of miR-146a, TNF receptor-associated protein 6 (TRAF6) and IRAK1, are downregulated by miR-146a when NF-*κ*B activity is reduced during inflammation, and in turn, expression of NF-*κ*B signaling downstream genes, such as IL-1*β*, IL-6, IL-8, and TNF*α*, is suppressed [56, 57].

## 7. Conclusion

Research in recent years has revealed the roles of ncRNAs as essential regulators of vital cellular processes under both normal and pathological conditions. However, our understanding of their functions as regulators of gene expression remains incomplete. Importantly, several ncRNAs have been identified that represent not only potential biomarkers for the diagnosis of AS in an early stage but also promising therapeutic targets for AS treatment. These ncRNAs include hsa-miR-29, hsa-miR-126-3p, and miR-196a [21, 26]. Moreover, both low-copy number ncRNA transcripts, e.g., miR-9-3, miR-143, miR-146a, miR-205, and high-copy number ncRNA transcripts, e.g., miR-23a and miR-301a, are associated with susceptibility to acute anterior uveitis combined with AS [27]. Additionally, miR-21 expression is closely associated with levels of programmed cell death 4 mRNA and collagen crosslinked C-telopeptide protein during AS progression [22].

lncRNA-AK001085 expression in serum is significantly decreased in AS patients compared with healthy individuals, indicating that it is a potential endogenous suppressor of AS. Notably, lncRNA-AK001085 expression can be induced by occupational hazards, cigarette smoking, and even lack of physical exercise. Based on the results of receiver operating curve analyses, lncRNA-AK001085 expression is an independent factor for the diagnosis of AS as well as in the BASFI test to determine disease activity [58].

Complex signaling networks including the Notch/Wnt, Hedgehog/Wnt, MAPK/Wnt, Wnt/BMP, Wnt/TNF, and TNF/NF-*κ*B pathways receive regulatory crosstalk from ncRNAs. ncRNA regulation of these pathways is essential in the process of new bone formation via inflammatory cascades. However, the roles of circular RNA and competing endogenous RNA networks remain to be investigated.

## Figures and Tables

**Table 1:** ncRNAs that are aberrantly expressed or have been shown to function in the pathogenesis of AS.

ncRNA	Signaling pathway(s)	Key signaling molecule(s)	Model(s)	Associated function(s)/mechanism(s)	Reference
miR-29a	Wnt	DKK1 and GSK3*β*	hFOB cells	Regulates TNF*α* and pathogenic bone metabolism	[9]
miR-29a	Wnt	DKK1	PBMCs	Diagnostic marker of new bone formation	[10]
miR-29a/miR-29c	Wnt	DKK1	osteoblasts	Post-transcriptional mechanisms for osteonectin regulation	[11]
miR-130a	TNF	HDAC3	PBMCs	HDAC3 forms a negative feedback loop with miR-130a and enhances TNF-1*α* expression	[12]
miR-10b	TNF/T cell-mediated prosurvival	IL-17A/MAP3K7	Th17 cells	miR-10b acts in a feedback loop to suppress IL-17A by targeting MAP3K7	[13]
lnc-ZNF354A-1/ lnc-LIN54-1/ lnc-FRG2C-3/ lnc-USP50-2	TGF*β*-BMPs	TGF*β*	MSCs	Pathological osteogenesis	[14]
hsa-miR-20a/ hsa-miR-300/ hsa-miR-185/ hsa-miR-30d/ hsa-miR-320a/ hsamiR- 130b/ hsa-miR-33a/ hsa-miR-155/ hsa-miR-222	TGF*β*-BMPs	BMP2/osteocalcin/ Runx2	osteoclasts/ fibroblasts	Regulation of cell-cell interaction between osteoclasts and fibroblasts	[15]
miR-199a-5p	mTOR/T cell-mediated prosurvival	Rheb	Th17 cell	Induces autophagy	[16]
miR-16/miR-221 /let-7i	T cell-mediated prosurvival	TLR-4/IFN-*γ*	T cells	Increased let-7i expression facilitates immune response	[17]
miR-124	T cell-mediated prosurvival	ANTXR2	Th1 cells	Induces autophagy	[18]
miR-155	NF-*κ*B	IFN-*β*	osteoclasts	Selectively interacts with both SOCS1 and MITF	[19]
miR-146a/ miR-155	—	—	serum samples	Novel complementary biomarkers	[20]
hsa-miR-29/ hsa-miR-126-3p	—	—	PBMCs	Biomarkers and provocative therapeutic targets	[21]
miR-21	—	PDCD4	whole blood	Development of AS	[22]

Abbreviations: PBMCs, peripheral blood mononuclear cells; MSCs, mesenchymal stem cells

**Table 2:** miRNAs mutations involved in the pathogenesis of AS.

ncRNA	SNP No.	Key signaling molecule	Associated or not	Associated function/mechanism	Reference
miR-146a	rs2910164	IRAK1 (rs3027898)	Yes	polymorphisms	[23]
miR-146a	rs2910164	—	Yes	polymorphisms	[24]
miR-499	rs3746444	—	No	polymorphisms	[24]
miR-146a	rs2431697/ rs2910164/ rs57095329	—	No	polymorphisms	[25]
miR-196a	rs11614913	Bach1, IL-1*β* and MCP-1	No	polymorphisms	[26]
miR-143/ miR-146a/ miR-9-3/					
miR-205/	—	—	Yes	copy number variants	[27]
miR-301a/					
miR-23a					
